# Effect of Awns on Photosynthesis and Yield of Triticale Under Water Deficit Compared to Flag Leaves

**DOI:** 10.3390/plants15040531

**Published:** 2026-02-08

**Authors:** Zhiling Lin, Wenhua Du

**Affiliations:** 1 Key Laboratory of Grassland Ecosystem (Ministry of Education), Pratacultural Engineering Laboratory of Gansu Province, College of Grassland Science, Sino-U.S. Centers for Grazing Land Ecosystem Sustainability, Gansu Agricultural University, Lanzhou 730070, China; 2 College of Forestry and Grassland Science, Jilin Agricultural University, Changchun 130118, China

**Keywords:** triticale (× *Triticosecale wittmack*), awn, spike photosynthesis, water deficit, grain-filling stage

## Abstract

Water deficit during the grain filling stage is a major threat to sustainable crop production, especially in arid and semi-arid regions. This study aims to investigate the comprehensive effects of water deficit on the photosynthetic characteristics, dry matter accumulation, and yield formation of triticale (× *Triticosecale wittmack*). The photosynthetic characteristics of the flag leaf and spike organs (awn, glume, and lemma) and their relationship with yield were analyzed during the grain filling stage when the triticale plant was subjected to adequate water supply and water deficit 0, 7, 14, 20, and 25 days after anthesis. The results showed that under normal water supply, photosynthesis was reduced by 27.5% and 34.4% in awned and awnless spikes, respectively, at 25 days after anthesis (25DAAs), compared to flag leaves. Under water deficit at 25DAAs, photosynthesis was reduced by 50.5% and 60.9% in awned and awnless spikes, respectively. Water deficit reduced RWC and chlorophyll content in flag leaf and spike organs, and the changes in RWC and chlorophyll content in spike organs were less than those in flag leaf. The differences in grain yield, biomass, and harvest index of awned and awnless triticale were not significant under adequate water supply. Grain yield of awned and awnless triticale was reduced by 23.7 and 24.6%, respectively, under water deficit compared to adequate water supply. Our results suggest that awnless plants suffer from drought more than awned plants in grain-filling stage, where the role of awns is critical.

## 1. Introduction

The growth and development of plants are often negatively impacted by environmental stresses, leading to reduced productivity. Among all environmental stresses, drought is considered the most destructive to plant productivity [1] and it is a critical factor constraining global agricultural development. Unlike most other natural disasters, drought can occur in any climatic region and has a broad spatial impact [2]. Influenced by rising global temperatures and climate change, the temporal and spatial distribution of precipitation events has become increasingly uneven [3,4]. As a result, the frequency and intensity of drought stress in arid and semi-arid regions have intensified. Moreover, the mismatch between the plant water demand period and the precipitation period often leaves plants under varying degrees of water stress [5]. This water stress can range from insufficient atmospheric moisture caused by low humidity to soil drought, thereby severely affecting plant growth, development, and yield formation.

Triticale (× *Triticosecale wittmack*) is a sexual hybridization of wheat (*Triticum aestivum* L.) and rye (*Secale cereale* L.) which combines the high quality of wheat and the large biomass and resistance of rye [6], a high-protein forage crop [7,8,9]. Due to its strong drought resistance and high biomass, it is popularly grown in northern China. In recent years, with global climate change, there have been frequent drought events in northern China [10], which may threaten future triticale production and breeding. In particular, droughts occurring during the filling stage can lead to severe reductions in photosynthesis and seed yield. Photosynthesis is an important physiological basis for the development of crop dry matter [11] and yield and is a key process in the response to drought stress [12]. Photosynthesis in plants is particularly sensitive to drought stress, leading to stomatal closure, reduced photosynthetic efficiency, and degradation of photosynthetic pigments [13]. Water deficit prior to fertility in wheat is not conducive to an increase in stomatal conductance [14]. Therefore, understanding the contributions of different photosynthetic processes in the photosynthetic organs of triticale to grain filling under drought stress is of great significance for triticale production.

The flag leaf is the primary photosynthetic organ in grasses. Spike organs (glumes, lemma, and awn) are important sources of carbohydrates and provide energy for grain filling through photosynthesis during the later stages of plant development [15,16]. Studies have shown that most of the carbon assimilated by the flag leaf is used for structural carbohydrates and tiller growth rather than being translocated to the grains [17,18]. Additionally, the flag leaf tends to wilt and senesce, while the spike has stronger vitality [19]. It can maintain a more intact ultrastructure, higher water use efficiency, and delayed senescence, with higher photosynthetic rates that are advantageous for grain filling under water-limited conditions [20,21].

After flowering, as the leaves progressively undergo senescence, non-leaf photosynthetic organs—particularly the triticale spike—assume a more significant role in the process of grain filling [22]. The panicle is an important photosynthetic organ in Gramineae (Poaceae), exhibiting stronger drought resistance and photosynthetic persistence than the flag leaf under drought conditions. The awn, as an important photosynthetic organ of the spike, makes a significant contribution to photosynthesis that cannot be ignored, accounting for 50% to 60% of the total photosynthetic area of the spike [23]. The awn facilitates the capture of light by cereal crops, thereby increasing the rate of photosynthesis [24,25]. The net photosynthesis of the barley spike can be attributed up to 90% to the awn [26]. Furthermore, the awn is responsible for approximately 50% of the net photosynthetic contribution to the grain within the spike [27]. The presence of awns has been demonstrated to increase grain size in studies on near-isogenic lines (NILs) of wheat. Nevertheless, the ultimate yield did not necessarily increase as a result of the larger grain size [27]. This may be attributed to the consumption of carbon assimilates by the growth of awns, which consequently reduces the allocation of assimilates available for floret primordia development. In contrast, the findings of Li et al. [14] indicate that the presence of awn plays a pivotal role in the germination process of wheat seeds. Following the removal of awn, the average weight of each seedling decreased by 32.2%. Consequently, further investigation is required to ascertain the impact of awn on the photosynthetic activity and yield of triticale in conditions of water deficit.

This study investigated the changes in photosynthetic physiological traits of different photosynthetic organs using a pair of near-isogenic lines (NILs) with and without awns under relative abiotic stress (water deficit) conditions. It preliminarily explored the pathways through which major photosynthetic organs contribute to grain yield. The study provides valuable evidence regarding spike characteristics, which can be considered as important indicators for breeding new materials of triticale in dryland areas and contribute to the theoretical basis for increasing triticale yield.

## 2. Materials and Methods

### 2.1. Plant Material and Experimental Method

The College of Grassland Science at Gansu Agricultural University provided awned near-isogenic line (NIL) materials of triticale. In 2013, the triticale hybrid line T17, which has excellent panicle traits and is awnless, was chosen as the female parent, and the high-biomass-yielding line J6 was chosen as the male parent. The F_1_ generation was obtained through hybridization. Subsequently, a stable, relatively pure, and harmonious recombinant inbred line (RIL) population was established through successive hybridization using the single-grain transmission method. From this advanced generation of isolated populations, a pair of awned near-isogenic line materials was successfully screened and identified.

We named the triticale materials as Y and W for awned and awnless materials, respectively, and the potting trial was conducted from October 2023 to February 2024 in a rain screen room at Gansu Agricultural University. The experimental setup utilized polyethylene plastic buckets with an upper mouth diameter of 23 cm, a lower mouth diameter of 16 cm, and a height of 18 cm. The soil used was yellow loam, sourced from the cultivated layer of the field (0–20 cm), which was air-dried and sieved. Each kilogram of soil was amended with 0.533 g of urea and 0.3 g of potassium dihydrogen phosphate. Prior to sowing, each pot was filled with 7.5 kg of soil, with an air-dried soil moisture content of 13.6%. Eighteen triticale seeds were sown per pot, and at the three-leaf stage of triticale, 10 seedlings of uniform growth were selected for retention, with any excess tillers being removed. The experiment was set up with two treatments, control (C) and water deficit (D), where soil moisture was maintained at 70–75% (C) and 40–45% (D) of the field water holding capacity, and the soil moisture content was 20.51–21.98% and 11.72–13.19%, respectively. In this experiment, water control was started 5 d before flowering, and water was replenished daily according to the water control standard by weighing method, so that the soil water content of each triticale pot reached the water control standard at flowering. On the flowering stage, the normal development and consistent size of the spike were selected and labeled.

### 2.2. Determination of Photosynthetic Parameters in Triticale Spike and Flag Leaf

A portable gas exchange photosynthesis system LI-6400XT (LI-COR, Inc., Lincoln, NE, USA) was employed to ascertain the net photosynthetic rate (Pn), transpiration rate (Tr), stomatal conductance (Gs), and intercellular CO_2_ concentration (Ci) of flag leaves and spikes. After calibrating the parameters of the photosynthesis meter, the temperature, light intensity, and CO_2_ concentration were set. Healthy leaves and panicles were selected for measurement. Once the readings stabilized, parameters were recorded [28]. Five biological replicates were established for each treatment. All measurements were operated between 9:00 am and 12:00 am on a sunny day at 0, 7, 14, 20, and 25 days after antithesis (DAAs), with a flow rate of 500 mL min^−1^ and a CO_2_ concentration of 420 μmol mol^−1^. Choose a red-blue light source leaf chamber and set light intensity at 1000 µmol·m^−2^·s^−1^ and leaf chamber temperature at 30 °C. The spike surface area was calculated in accordance with the equation proposed by Zhang et al. [29,30]. The total surface area of the spike was calculated using the following formula: Spike length × Spike width × 3.8 (glume surface area) + Total awn length of the top third spikelet × Number of spikes × 0.1 (awn surface area). This was performed for three biological replicates and three technical replicates.

### 2.3. Determination of Chlorophyll (Chl) in Triticale Spike and Flag Leaf

Chlorophyll content was determined by the method of Gorooei et al. [31]. Flag leaves, glumes, lemmas, and awns were cut into small pieces and soaked in 25 mL of 80% acetone immediately after harvesting and placed in a dark space for about 2 d until the samples turned white. The absorbance of the extracted solution was measured with spectrophotometer (TU-1901, Beijing Puxi General Instrument Co., Ltd., Beijing, China) at 663 nm, 645 nm, and 470 nm, and 80% acetone was used as a blank control. Three biological replicates were set up for each treatment.

*Chla* = 12.21 × *A*_663 − 2.81 × *A*_645

*Chlb* = 20.13 × *A*_645 − 5.03 × *A*_663

*Chl* = *Chla* + *Chlb* = 20.29 × *A*_645 + 8.05 × *A*_663

### 2.4. Determination of Relative Water Content (RWC) in Triticale Spike and Flag Leaf

RWC was determined according to Abebe et al. [32]. Glumes, lemma, and palea were first weighed for fresh weight (FM), then the samples were soaked in distilled water overnight at 4 °C and weighed (TM). The leaves were then dried at 80 °C for 24 h to obtain the final dry mass (DM). Six biological replicates were analyzed. RWC was calculated according to Equation:

*RWC* (%) = (*FM* − *TM*)/(*TM* − *DM*) × 100%

### 2.5. Determination of Yield

A selection of 10 triticale plants were harvested at maturity with the aim of determining the biomass of triticale. In addition, in each pot, the composition of the yield of triticale was investigated to determine the number of spikelets, the number of grains per spike, and the thousand-grain weight. Harvest index (HI) of triticale was also calculated:

*HI* = (*Grain yeild*)/*Biomass*

### 2.6. Statistical Analysis

The data were calculated and plotted using Excel 2021 and then processed using SPSS 22.0, with results presented as the standard error of the mean based on three replicates. Duncan’s multiple range analysis at *p* < 0.05 was used to detect significant differences by using SPSS statistical software. The correlation heatmap was generated using the “ggplot2” package in R software (v4.5.2). Partial Least Squares Path Modeling (PLS-PMs) was employed to evaluate the direct and indirect effects of the photosynthetic physiological traits of the flag leaf and awn on grain yield, with the model executed using the R package “plspm” (v4.5.2).

## 3. Results

### 3.1. Effect of Water Deficit on Net Photosynthetic Rate and Transpiration Rate of Spike and Flag Leaf

**Figure 1:**
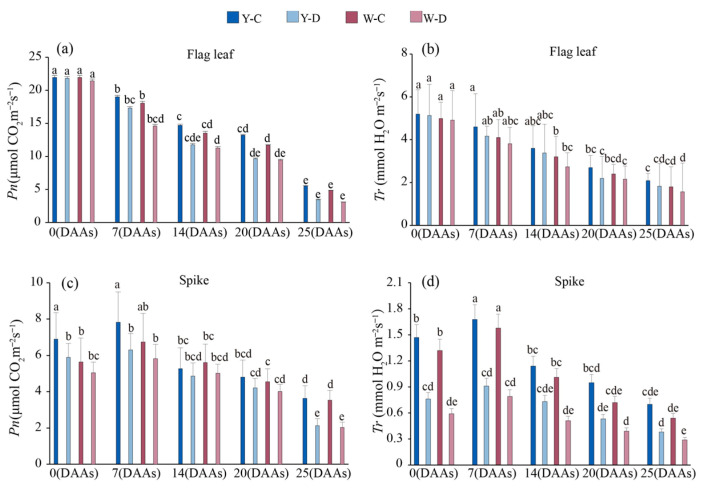
Net photosynthetic rates and transpiration rate of flag leaf (**a**,**b**) and spike (**c**,**d**) ofawned (Y) and awnless (W) triticale under control (C) and water deficit (D) conditions. Lowercase letters represent significance at *p* < 0.05 among different treatments. DAAs—days after anthesis.

Flag leaf net photosynthetic rate (Pn) and transpiration rate (Tr) varied consistently and without significant differences between awned and awnless triticale during the filling stage. The flag leaf Pn decreased at a faster rate under the water deficit treatment compared to the normal water supply (Figure 1a). However, under all conditions, the Pn of awned triticale spikes showed an increasing and then decreasing trend (Figure 1c), reaching a peak at 7DAA. Compared with the normal water supply, the average Pn of the flag leaves of the two NIL triticale decreased by 20.0% and 16.8% at 14 and 20 DAA in the water deficit (Figure 1a), while it decreased by 0.42 and 0.59 μmol CO_2_ m^−2^ s^−1^ in the awned spikes and 0.60 and 0.53 μmol CO_2_ m^−2^ s^−1^ in the awnless spikes (Figure 1c), respectively. From 14 DAAs to 25 DAAs, the Pn of awned triticale spikes decreased by 1.65 and 2.08 μmol CO_2_ m^−2^ s^−1^ under the two different water conditions, respectively. Meanwhile, the Pn of awnless triticale spikes decreased by 2.72 and 2.98 μmol CO_2_ m^−2^ s^−1^ under the two different water conditions, respectively (Figure 1c). The Tr of flag leaf and spike showed a similar trend to that of Pn (Figure 1b), and spike Tr peaked 7DAA (Figure 1d). Compared with the normal water supply treatment, Tr of flag leaves of the awned NIL decreased by 0.30 mmol H_2_O m^−2^ s^−1^ at 25 DAAs in the water deficit treatment (Figure 1b), while Tr of both awned and awnless spikes decreased by 0.32 and 0.25 mmol H_2_O m^−2^ s^−1^ (Figure 1d).

### 3.2. Water Deficit on Stomatal Conductance of Spike and Flag Leaf

**Figure 2:**
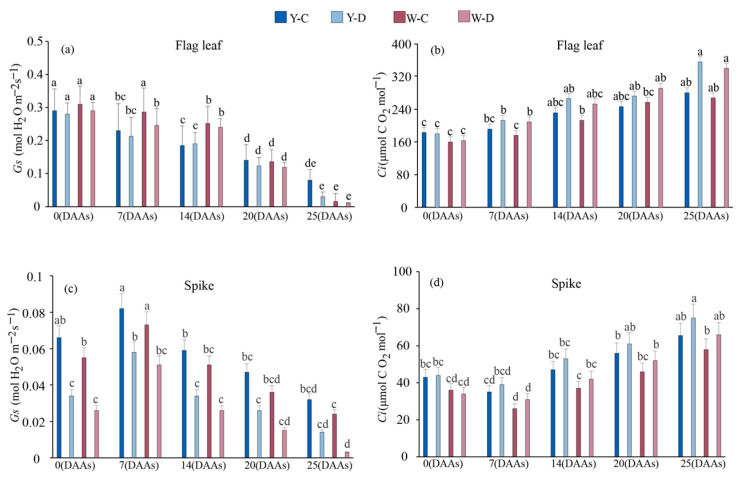
Stomatal conductance and intercellular CO_2_ concentration of flag leaf (**a**,**b**) and spike (**c**,**d**) of awned (Y) and awnless (W) triticale under control (C) and water deficit (D) conditions. Lowercase letters represent significance at *p* < 0.05 among different treatments simultaneously. DAAs—days after anthesis.

Compared with normal water supply, the mean Gs of NILs flag leaves decreased by 0.015, 0.029, 0.004, 0.017, and 0.028 (mol H_2_O m^−2^ s^−1^) at 7, 14, 20, and 25 DAAs, under water deficit, respectively (Figure 2a). Awnless spikes showed higher decreases in the water deficit compared with awned spikes. At 25 DAAs, under normal water supply and water deficit, the Gs of the awned triticale spike was 0.33 and 3.67 times higher than that of the awnless triticale spike (Figure 2c). Under water deficit conditions, the decrease in Gs in awned triticale spikes from 0 to 25 DAAs was 34.16% lower than that in the flag leaves; the decrease in Gs in awnless triticale spikes during the same period was only 4.52% different from that in the flag leaves. Ci in both the flag leaf and spike showed an upward trend during grain filling. Under water deficit conditions, the flag leaf Ci of awned triticale increased to a greater extent than that of awnless triticale during the period of 0 DAAs to 14 DAAs (Figure 2b,d). Under water deficit conditions, from 0 to 25 DAAs, the increase in Ci in awned triticale spikes was 27.32% lower than that in the flag leaves. In contrast, the increase in Ci in awnless triticale spikes during the same period was 14.47% different from that in the flag leaves.

### 3.3. Effect of Water Deficit on Relative Water Content of Spike and Flag Leaves

**Figure 3:**
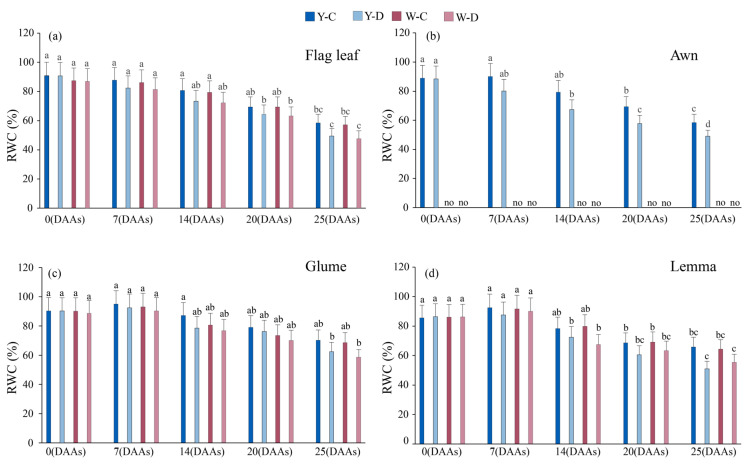
Relative water content of flag leaf and spike of awned (Y) and awnless (W) triticale under control (C) and water deficit (D) conditions. (**a**), flag leaf; (**b**), awn; (**c**), glume; and (**d**), lemma. Lowercase letters represent significance at *p* < 0.05 among different treatments simultaneously. “no” denotes the absence of data on the awns of awnless triticale. DAAs—days after anthesis.

Compared with normal irrigation, the RWC of the organs (glumes, lemma, awn, and flag leaf) in both awned and awnless triticale significantly decreased under water deficit conditions (Figure 3). In awned triticale, at 25 DAAs under water deficit, the RWC in the glumes, lemma, awn, and flag leaf decreased by 9.78%, 7.8%, 10.59%, and 10.4%, respectively, compared with normal water supply. In awnless triticale, at 25 DAAs under water deficit, compared with normal water supply, the value of RWC decreased by 3.96% in the glume, 16.06% in lemma, and 8.68% in flag leaf. In awned triticale, under water deficit conditions, the RWC of the awns showed a lower decrease than that in the flag leaves from 0 to 25 DAAs. In awnless triticale, under water deficit conditions, the RWC in the glumes decreased more than that in the flag leaves during the same period.

### 3.4. Effect of Water Deficit on Chlorophyll Content of Spike and Flag Leaves

**Figure 4:**
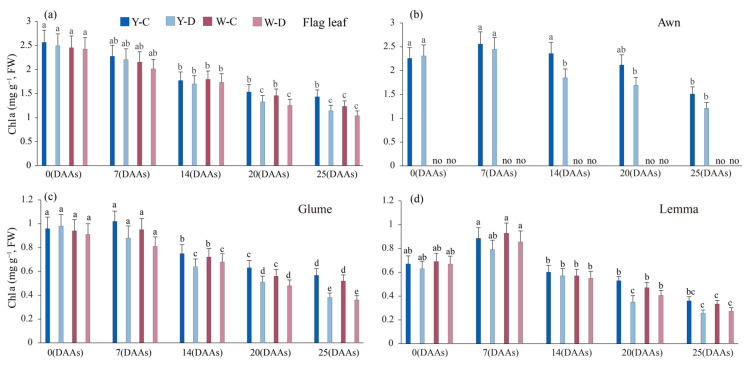
*Chla* content of flag leaf and spike of awned (Y) and awnless (W) triticale under control (C) and water deficit (D) conditions. (**a**), flag leaf; (**b**), awn; (**c**), glume; and (**d**), lemma. Lowercase letters represent significance at *p* < 0.05 among different treatments simultaneously. “no” denotes the absence of data on the awns of awnless triticale. DAAs—days after an-thesis.

The Chla content of the awned NIL spike (awn, glumes, and lemma) first increases and then decreases, except for that in the awned NIL glumes under water deficit conditions, which gradually decreased with time (Figure 4). The Chla content of awned triticale spikes (awns, glumes, and lemmas) decreased less than that in awnless triticale spikes (glumes, lemmas) under water deficit conditions during the period of 0 DAAs to 25 DAAs. At 25 DAAs, the Chla content of glumes and lemmas decreased by 0.12 and 0.09 mg g^−1^ (awned) and 0.09 and 0.09 mg g^−1^ (awnless), respectively, under water deficit conditions. Compared with 0 DAAs, at 25 DAAs, the Chla content in the flag leaves in both awned and awnless NILs decreased by 1.13 and 1.29 mg g^−1^ under normal water supply, and by 1.13 and 1.32 mg g^−1^ under water deficit, respectively.

**Figure 5:**
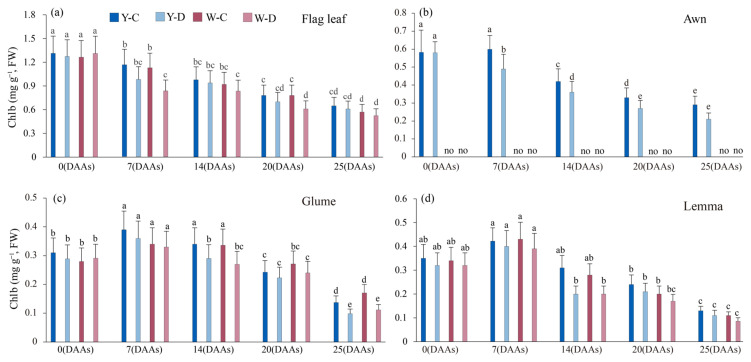
*Chlb* content of flag leaf and spike of awned (Y) and awnless (W) triticale under control (C) and water deficit (D) conditions. (**a**), flag leaf; (**b**), awn; (**c**), glume; and (**d**), lemma. Lowercase letters represent significance at *p* < 0.05 among different treatments simultaneously. “no” denotes the absence of data on the awns of awnless triticale. DAAs—days after anthesis.

For Chlb, the trend of the different treatments from 0 DAAs to 25 DAAs was consistent with that of Chla, except for glumes of water deficit-treated awned NIL, and water deficit also had an inhibitory effect on Chlb content (Figure 5). Compared to normal water supply, the Chlb content of glumes and lemmas decreased by 0.02 and 0.07 mg g^−1^ (awned) and 0.02 and 0.12 mg g^−1^ (awnless), respectively, under water deficit, at 25 DAAs (Figure 5c,d).

**Figure 6:**
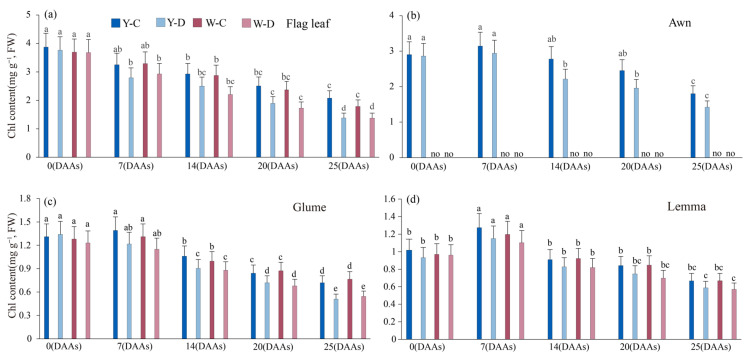
Total *Chl* content of flag leaf and spike of awned (Y) and awnless (W) triticale under control (C) and water deficit (D) conditions. (**a**), flag leaf; (**b**), awn; (**c**), glume; and (**d**), lemma. Lowercase letters represent significance at *p* < 0.05 among different treatments simultaneously. “no” denotes the absence of data on the awns of awnless triticale. DAAs—days after anthesis.

Compared with the flag leaf, the rate of decline in total Chl content in the spikes (awns, glumes, and lemmas) was relatively slower, especially under water deficit conditions (Figure 6b). The total Chl content in the flag leaves of NILs showed the same trend as the Chla content with the increase in days after anthesis, under both conditions, but the degree of decrease was different. Compared with awned triticale, the rate of decline in total Chl content in the flag leaf of awnless triticale is higher. Compared with normal water supply, at 25 DAAs, under water deficit conditions, the total Chl content in the flag leaf, awn, glume, and lemma of awned triticale decreased 0.11 mg g^−1^, 0.38 mg g^−1^, 0.21 mg g^−1^, and 0.09 mg g^−1^, respectively; the total Chl content of awnless triticale decreased by 0.11 mg g^−1^ in flag leaf, 0.19 mg g^−1^ in glume, and 0.15 mg g^−1^ in lemma (Figure 6a–d). In both awned and awnless triticale, the total Chl content of the spike organs (awns, glumes, and lemma) decreased less than that of the flag leaves from 0 to 25 DAAs under water deficit conditions.

### 3.5. Effect of Water Deficit on Biomass and Yield Components

**Figure 7:**
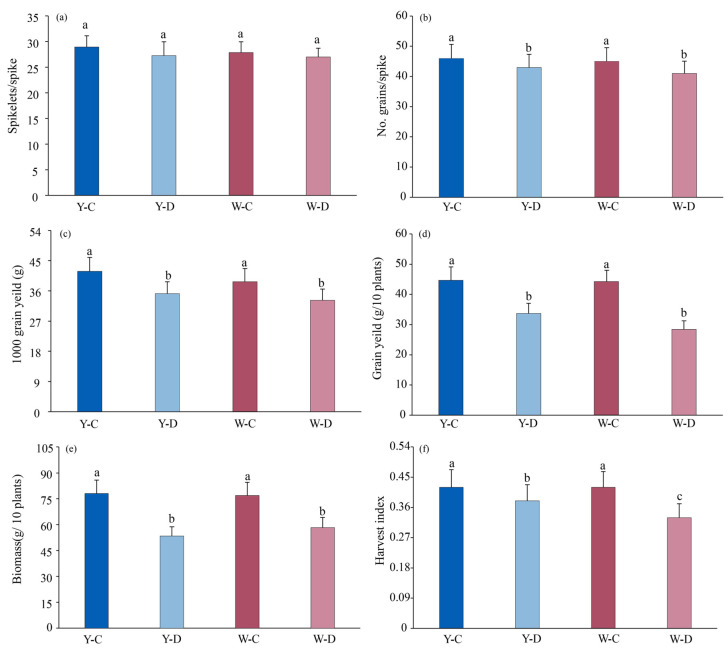
Triticale yield and yield components of awned (Y) and awnless (W) triticale under control (C) and water deficit (D) conditions. (**a**) Spikelets; (**b**) No. grains; (**c**) 1000 grain yield; (**d**) Grain yield; (**e**) Biomass; (**f**) Harvest index. Lowercase letters represent significance at *p* < 0.05 among different treatments simultaneously.

Water deficit significantly affected grain yield and grain yield components (No. grains/spike, 1000 grains yield) of NILs triticale (Figure 7b–d). Under water deficit, grain yield was slightly lower in awnless than in awned triticale. Compared to normal water supply, the number of grains were significantly lower in awned and awnless triticale under water deficit; the number of grains decreased in awned (6.5%) and awnless (8.9%) triticale spikes. The thousand kernel weight of awned (14.4%) and awnless (15.8%) triticale decreased under water deficit compared to normal water supply.

There were differences in biomass and harvest index among different awned triticale under water deficit (Figure 7e,f). Compared to the normal water supply, the biomass and harvest index decreased under water deficit. Compared with normal water supply, the biomass of awned triticale and awnless triticale decreased by 31.5% and 24.1% under water deficit conditions. Under water deficit, the harvest index of both awned and awnless triticale was significantly lower than that under normal water supply.

### 3.6. The Correlation Between Photosynthetic Characteristics, Grain Yield, and Biomass

**Figure 8:**
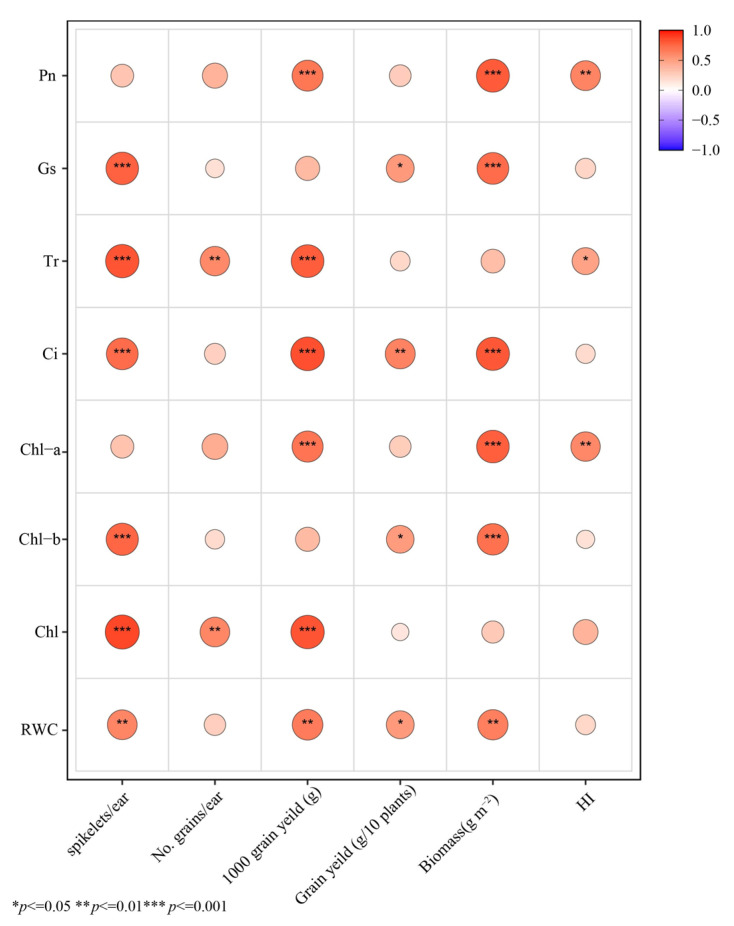
Correlation between photosynthetic characteristics, RWC, and yield components of triticale under water deficit.

Pearson correlation analysis indicates (Figure 8) that most physiological traits are positively correlated with yield and yield component traits overall, and many trait combinations reach statistical significance (*p* ≤ 0.05, *p* ≤ 0.01, and *p* ≤ 0.001), with almost no obvious negative correlations observed. Regarding yield-related traits, the 1000 grain yield shows the most pronounced correlations with multiple physiological indicators: Pn, Tr, Ci, Chl-a, and total chlorophyll (Chl) all exhibit significant positive correlations. Meanwhile, biomass is also significantly positively correlated with Pn, Gs, Ci, and chlorophyll-related indices such as Chl-a and Chl-b.

For spike traits, spikelets per ear are significantly positively correlated with Gs, Tr, Ci, and some chlorophyll indices (Chl-b and Chl). In contrast, the number of physiological traits significantly associated with grain yield is relatively limited, but grain yield still shows significant positive correlations with Ci, Gs, Chl-b, and RWC. The harvest index (HI) shows generally weak correlations with most physiological traits, with significant positive correlations observed only for Pn, Tr, and Chl-a.

### 3.7. The Role of Photosynthetic Characteristics of Flag Leaves and Different Awn Types on Yield in Triticale

**Figure 9:**
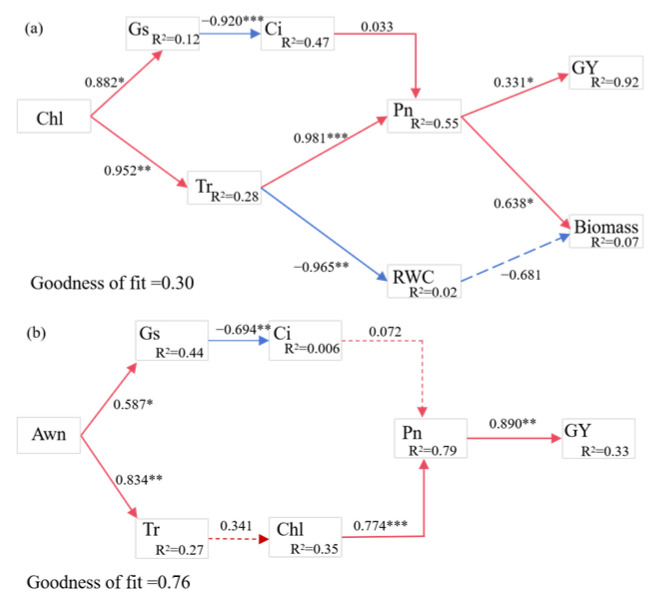
The correlation between photosynthetic characteristics, grain yield, and biomass of flag leaf (**a**) and awn (**b**) by partial least squares-path modeling (PLS-PM). Red means positive impacts, while blue shows negative impacts. A solid line represents significant effects, while dotted line represents non-significant effects. * *p *≤ 0.001; ** *p *≤ 0.01; *** *p *≤ 0.05.

The partial least squares-path modeling (PLS-PM) analysis shows that flag leaves and awns of triticale differ in their pathways of action on photosynthetic characteristics and seed yield during the filling stage of triticale. Chlorophyll content in flag leaves significantly contributed to Gs and Tr, and Gs negatively regulated Ci. Ci and Tr positively regulated Pn of flag leaves, then significantly increased grain yield and biomass (Figure 9a).

The presence or absence of awns significantly increased Tr and Gs. Tr promoted chlorophyll content and increased Pn in spike; Ci is negatively regulated by Gs but has a positive regulatory effect on the number of grains per spike, and in turn, it significantly increased grain yield (Figure 9b).

## 4. Discussion

### 4.1. Effect of Awns on Photosynthesis of Triticale Under Water Deficit

Stomatal opening promotes leaf photosynthesis, and stomata are a major defense against drought, with plant survival dependent on stomatal closure under drought stress [33,34]. Zhou et al. [35] showed that drought stress leads to a reduction in stomatal conductance, photosynthetic rate, and chlorophyll content of soybean plants. Severe drought stress leads to increased water loss from leaves, resulting in shortened stomatal length [36]. In this study, we observed a severe reduction in stomatal conductance with increasing water deficit time in the flag leaf of triticale, with relatively little effect on glumes, lemmas, and awns (Figure 2). We also found that Pn and Tr decreased less in the spike than in the flag leaf under drought conditions (Figure 1). These findings suggest that photosynthesis is reduced to a lesser extent in the spike than in the flag leaf in stressed triticale. The importance of awns is similar to that found for flag leaves, especially awned species with strong photosynthetic capacity [37,38]. The awn organ, which plays a key role in the filling stage, has also been observed in other grass species, such as barley. Comparative transcriptional analyses also showed that the long awn is the main photosynthetic organ of the spike [39,40]. In this study, Pn in awned triticale spikes under water deficit decreased less than that in awnless spikes in the late stage of filling (14DAA-25DAA) (Figure 1c), which also verified the above observation.

RWC is a specific expression of water status and can be used as one of the indicators of drought tolerance in cereal crops. The better water retention capacity of awns compared to flag leaves may be one of the reasons for their higher photosynthetic efficiency under water deficit (Figure 1 and Figure 3). Under water deficit conditions, the increase in Ci in the spike is lower than that in the flag leaves as days after anthesis extends. Moreover, the Ci in awnless spikes is lower than that in awned spikes. This may be because the awnless spikes rapidly close their stomata, thereby directly limiting the entry of external CO_2_ into the spike, while the photosynthesis of the awn provides additional CO_2_ fixation [41]. It indicated that drought stress inhibited photosynthesis in triticale, and the awn had better drought tolerance than the flag leaf and could better carry out carbon assimilation under water deficit, which was similar to some previous reports [42,43]. In the study, awns of triticale are able to maintain higher RWC than flag leaves under drought stress [34]. Among the plant organs studied, awns have the most pronounced thick-walled tissue structure, which makes them tolerant to drought stress [44], and therefore maintained a more stable photosynthesis compared to the flag leaf.

Reduction in chlorophyll content is considered to be a typical marker of photooxidation and chlorophyll degradation [45]. Under drought stress conditions, leaf chlorophyll content is reduced, which disrupts photosynthesis [46] and chlorophyll pigment degradation due to stress-induced metabolic imbalance. In the present study, water deficit resulted in reduced chlorophyll content (Figure 2a–d). The chlorophyll content decreased linearly with increasing duration in water deficit. In addition, total Chl content decreased more in the flag leaf than in the spike during the filling stage as a result of nutrient translocation and leaf senescence [47], indicating that the photosynthetic spikelet was less sensitive to water loss, suggesting that spikelet organs were less affected by water loss and thus maintained more stable photosynthesis compared to flag leaves. It was also shown that water deficit decreased Chla and Chlb content in awned triticale spikes more than awnless spikes, increasing the absorption of short-wave light, which improves assimilate synthesis at the reproductive growth stage [14]. These results are in agreement with Swapna et al. [48], who stated that chlorophyll and carotenoid contents in rice plants decrease during drought stress. Drought stress resulted in the rate of photosynthesis, water content, and transpiration rate also associated with increased stomatal resistance [49].

### 4.2. Effect of Awns on Yield of Triticale Under Water Deficit

Under well-watered conditions, the awned and awnless materials showed no significant differences in spikelets per ear, grains per ear, 1000-grain weight, grain yield per plant, biomass, or HI (Figure 7), indicating that water regime was the dominant driver of variation. Under drought, grains per ear, 1000-grain weight, grain yield, and biomass declined markedly in both materials (Figure 7), suggesting that yield loss resulted from a combined limitation of sink establishment (reduced grain number), grain filling (reduced grain weight), and assimilate accumulation (reduced biomass). This pattern aligns with the canonical cereal response to water deficit, where drought around heading/flowering impairs floret fertility and grain set, while drought during grain filling restricts photosynthesis and assimilate supply, thereby reducing grain weight [50]. This might be attributed to drought stress reducing leaf water status and stomatal conductance (characterized by decreased relative water content and stomatal conductance), intensifying CO_2_ diffusion limitation, and suppressing net photosynthetic rate (Figure 1 and Figure 2). Consequently, this led to reduced biomass accumulation and insufficient assimilate supply for grain filling, thereby resulting in a decrease in 1000-grain weight.

Notably, the absence of yield advantage of awned triticale over awnless triticale under the same water regime is more likely attributable to the environmental dependency of awn effects and source–sink trade-offs. Under non-stressful agronomic conditions, grain production often approaches a sink-limited state rather than a source-limited state. Consistently, under control conditions, both grains per ear and 1000-grain weight were similar between the two materials (Figure 7), implying that even if awns provide extra photosynthetic area, the incremental assimilate gain may not translate into yield when grain-filling potential is already close to its upper limit. Previous studies have shown that spike photosynthesis (including awns) can contribute carbon for grain filling, but its relative contribution depends on environment and the progression of organ senescence; under favorable conditions, leaves (especially the flag leaf) remain major sources, and the contribution of awns may be insufficient to produce a detectable yield advantage [41].

Furthermore, why did awned accessions exhibit higher photosynthetic indices under drought conditions without a significant increase in yield? The key reason is that the yield limitation caused by drought often occurs during developmental stages where the compensatory effect of awns cannot offset such constraints. In Figure 7, drought significantly reduced grains per ear, indicating that sink capacity had already been compromised around flowering. By contrast, awn and spike photosynthesis mainly contribute during the post-anthesis grain-filling stage by supplementing assimilates to sustain grain filling and increase grain weight. Once grain number is substantially reduced, even if awns enhance the relative source supply or promote assimilate partitioning to grains (reflected as higher HI), this may improve partitioning efficiency but cannot overcome the dual constraints of reduced total biomass and reduced sink capacity, resulting in non-significant differences in final grain yield. In addition, awns may exert bidirectional effects on spike energy balance as follows: under hot and dry conditions, awns could alter spike temperature and transpiration–hydraulic processes, and their photosynthetic gains may be partly offset by respiratory costs, heat stress, or hydraulic limitation, leading to a neutral net yield effect [44,51]. Therefore, the pattern observed here is more consistent with awns stabilizing partitioning under stress (higher HI), but insufficient to compensate for the losses in grain number and biomass under the drought intensity and timing imposed in this experiment.

### 4.3. Relationship Between Photosynthetic Physiology and Yield Components Under Water Deficit Conditions

The correlation heatmap suggests that yield improvement is more likely closely associated with source strength and assimilate accumulation within the source–sink relationship. Chl-a and Pn show extremely significant correlations with 1000-grain weight and biomass (*p* < 0.001) and significant correlations with HI (*p* < 0.05). This indicates that higher light-harvesting capacity and photochemical potential may enhance dry matter accumulation and improve allocation efficiency to grains by sustaining higher photosynthetic production and assimilate supply during grain filling. When net leaf assimilation capacity increases, assimilating availability per grain becomes more sufficient, leading to higher 1000-grain weight and, consequently, greater population-level dry matter accumulation (Figure 8).

In contrast, Gs and Tr are more strongly associated with spike-related traits. Both are extremely significantly positively correlated with spikelets per ear and Gs shows a significant correlation with grain yield per plant (*p* < 0.01). Meanwhile, Tr reaches significant or extremely significant correlations with grain number per ear (*p* < 0.001). These results imply that during critical periods determining “potential sink capacity” (e.g., young spike differentiation and the stages around flowering), stomatal regulation and transpiration may indirectly promote spike differentiation and the establishment of grain set by influencing carbon assimilation as well as canopy energy and water balance (Figure 8).

The significant correlations of RWC with grain yield, 1000-grain weight, and biomass further indicate that higher tissue relative water content may help maintain stomatal openness and leaf physiological activity, thereby reducing assimilation loss and the risk of premature senescence under stress. This, in turn, supports sink filling while contributing to yield through biomass accumulation as the material basis.

Notably, some photosynthetic physiological traits show weaker correlations with grain number per ear than with 1000-grain weight and biomass, suggesting that under the conditions of this experiment, yield variation may be driven more by grain filling and assimilate accumulation (grain weight and total biomass) rather than grain number alone (Figure 8).

Together with the PLS-PM results, water-status maintenance (RWC) appears to safeguard the relative stability of stomatal regulation (Gs, Tr) and intercellular CO_2_ concentration, thereby enhancing assimilated supply and material accumulation during grain filling, which increases grain weight and allocation efficiency and ultimately leads to higher yield. In other words, the key pathway revealed by the model centers on photosynthesis-driven dry matter production and allocation efficiency, conferring an advantage during the grain-filling stage.

All the results indicated that the spike has a better ability to cope with water deficit than the flag leaf, resulting in relatively higher photosynthesis. Awned triticale had a yield advantage over awnless triticale under water deficit conditions. This was mainly due to the ability of awns to reduce the rate of chlorophyll loss and delay senescence, while awns increased light interception at the spike, and higher stomatal conductance promoted stronger photosynthesis, which in turn increased grain yield at the filling stage (Figure 9). Awns have a slight advantage under water deficit. It is commonly believed that awns are one of the last green tissues to develop on the plant and the last to senesce [52], which is the main reason why awns are commonly found to benefit yields in drought areas during irrigation. In our study, awns yellowed later than the rest of the spike, and yellowing was only slightly delayed in the rest of the spike compared to the flag leaf. If this is the case, selection for late awn senescence may produce genotypes in which canopy photosynthesis is maintained for a longer period of time than in awnless genotypes, resulting in a longer grain filling stage and, hence, higher grain yields.

## 5. Conclusions

In summary, compared to the flag leaf, the photosynthetic organs of the spike may exhibit delayed senescence. Awned triticale demonstrates higher and more stable photosynthetic performance than awnless triticale, especially under water deficit conditions. This finding provides reliable evidence regarding the photosynthetic persistence and delayed senescence of the spike and lays a theoretical foundation for exploring the potential for increased yield of triticale in semi-arid regions.

## Data Availability

The raw data supporting the conclusions of this article will be made available by the authors on request.
